# Carbapenemases in Gram-Negative Bacteria: Laboratory Detection and Clinical Significance

**DOI:** 10.1155/2014/841951

**Published:** 2014-06-15

**Authors:** Branka Bedenić, Vanda Plečko, Sanda Sardelić, Selma Uzunović, Karmen Godič Torkar

**Affiliations:** ^1^Clinical Department for Clinical and Molecular Microbiology, University Hospital Center Zagreb, 10000 Zagreb, Croatia; ^2^Department of Microbiology, School of Medicine, University of Zagreb, 10000 Zagreb, Croatia; ^3^Department of Microbiology, University Hospital Center Split, 21000 Split, Croatia; ^4^Cantonal Public Health Institute Zenica, 72000 Zenica, Bosnia and Herzegovina; ^5^Department for Sanitary Engineering, Faculty of Health Sciences, 1000 Ljubljana, Slovenia

Carbapenems are potent *β*-lactam antibiotics used to treat serious infections in hospital settings. In comparison to penicillins, cephalosporins, or *β*-lactam/*β*-lactamase inhibitor, they have broad antimicrobial spectrum that includes Gram-positive (e.g., imipenem, doripenem) and Gram-negative bacteria (e.g., meropenem, ertapenem). Imipenem and meropenem have better activity against* P. aeruginosa*, while imipenem and doripenem have better activity than meropenem against* Acinetobacter baumannii*. Doripenem has the lowest MIC against* P. aeruginosa* and* A. baumannii* in comparison to imipenem and meropenem, and it is least susceptible to hydrolysis by carbapenemases.

To act on PBPs, carbapenems have to enter the wall of Gram-negative bacteria through outer membrane proteins (porins). Binding to different PBPs, they inhibit the synthesis of cell wall finally leading to the death of bacterium [1].

Carbapenem resistance in Gram-negative bacteria can be the consequence of the production of a *β*-lactamase, expression of efflux pumps, porin loss, and alterations in PBPs. Since *β*-lactams, including carbapenem-like compounds, are natural products of several environmental bacteria and fungi, it is supposed that other bacteria started to produce their intrinsic *β*-lactamase to give them selective advantage for survival. Thus, several genes encoding different carbapenemases can be found in environmental bacteria like* Bacillus anthracis*,* Serratia fonticola*,* Pseudomonas cepacia*, or* Acinetobacter* spp. as part of their chromosome [1, 2]. Further step in this evolution of resistance was the escape of carbapenemase encoding genes to mobile genetic elements (plasmids, transposons) providing possibility of successful horizontal spread of resistance genes even between different genera [3].

Since this discovery, carbapenemases became a global problem. According to the Ambler classification (based on structural similarities), they belong to the classes A, B, and D [1]. Class A carbapenemases contain serine at their active site and are capable of hydrolyzing all *β*-lactams, including aztreonam. In this group of carbapenemases, Sme (Sme-1 to Sme-3), IMI (IMI-1 to IMI-3), NmcA, and SFC-1 enzymes are mostly chromosomally encoded, while KPC (KPC-2 to KPC-13) and GES (GES-1 to GES-20) are plasmid encoded. Dominant carbapenemase from this group is KPC, identified in 1996 in North Caroline, USA, now causing many regional outbreaks, with endemicity in northeastern part of the USA, Israel, China, Porto Rico, Colombia, and Greece, and becoming more and more prevalent throughout Europe [4]. Beside* K. pneumoniae*, represented by a predominant clone (ST258), it has been found in other Enterobacteriaceae, as well as in* P. aeruginosa *and* A. baumannii-calcoaceticus* complexes. It is sometimes difficult to be recognized since MICs to carbapenems are in many cases lower than the breakpoints [2, 5]. Class B carbapenemases are also known as metallo-*β*-lactamase (MBL) since they contain metal ion(s) in their active site. Beside those chromosomally located in environmental bacteria (*Bacillus cereus*-BCI, BCII,* Aeromonas* spp.-CphA, and* S. maltophilia-*L1), acquired MBL encoding genes are often located in gene cassettes within integron, being part of a plasmid or chromosome. Firstly described acquired MBLs were in Japan in 1991, so called IMP-enzymes (there are now more than 30 derivatives), and are still dominant MBLs in Asian continent causing mainly sporadic outbreaks [6]. VIM-enzymes (there are now more than 30 derivatives) were firstly described in* P. aeruginosa* but later emerged in Enterobacteriaceae as well and fastly spread over whole Europe, causing outbreaks in many Mediterranean countries (like Greece, Italy, and Turkey). VIM metallo-*β*-lactamase is now the most prevalent carbapenemase spreading globally and, although largely connected to* P. aeruginosa*, is now reported more often from Enterobacteriaceae from Mediterranean countries, particularly Greece and Turkey, with the description of many panresistant strains [6, 7]. Another worrisome metalloenzyme arose from India in 2008, namely, New-Delhi MBL (NDM-1; until now more than ten variants are described) and spread fastly over Indian subcontinent in the following few years. NDM-enzymes are mostly not only associated with nonclonally related isolates of* K. pneumoniae* and* E. coli* but also described in* P. aeruginosa *and* A. baumannii *[8]. Beside proven facts that those enzymes exist in isolates spreading in environment and are carried in general population by enteric flora, the magnitude of the problem potentiates the huge population reservoir from Indian subcontinent and Middle Asia that moves across the world spreading further the resistance genes [9–11]. Another new source of those enzymes could be the Balkan region [12, 13]. Oxacillinases from molecular class D demonstrating carbapenemase activity are often found in* Acinetobacter *spp. They are divided into the most globally spread OXA-23 group, found also in environmental isolate of* Acinetobacter *spp. suggesting the possible natural and not nosocomial source of these genes, OXA-24 group, not so widespread as OXA-23, mostly described in Europe and USA, and OXA-58 group, described in several outbreaks all over the world [14]. The problem became more global with the discovery of OXA-48 in Enterobacteriaceae, particularly in* K. pneumoniae* and to lesser extent in* E. coli*, spreading all around the world but specifically in countries close to the Mediterranean Sea [14–16].

Carbapenemase producing Gram-negative bacteria can cause a wide spectrum of infections including bacteraemia, nosocomial pneumonia, wound infections, endocarditis, and urinary tract infections. Those infections are often associated with treatment failures, long hospital stay, and high mortality rates; for example, attributable mortality for carbapenem resistant* P. aeruginosa* infections ranged between 51.2% and 95% [17, 18].

Ideally, methods for determining carbapenemase should have a short turn-around time to ensure timely implementation of control measures. This could be challenged by difficulties in detecting carbapenemase producers, since MICs to carbapenems could be elevated but within susceptible range or even low, as described in Enterobacteriaceae and* A. baumannii* [19].

However, relevant methodology with specific laboratory test has not yet been standardized. Modified Hodge test is the only test recommended by CLSI for the phenotypic detection of carbapenemase producers but often lacks sensitivity and specificity. There are also several inhibitor based tests using different inhibitors (EDTA and phenanthroline as inhibitors of MBLs, phenylboronic acid as inhibitor of KPC) in combination with carbapenem (e.g., meropenem) or cephalosporin (e.g., ceftazidime) in different format-disk diffusion or broth dilution or *E*-test [19].

There is no specific inhibitor that could be used in detection of class D carbapenemases, but there are reports on using temocillin disk (or combined with avibactam) for this purpose [20].

Carba NP test is a simple biochemical test based on hydrolysis of imipenem detectable by a change of colour of indicator due to decrease of pH. It is applicable in most microbiological laboratories, although the reference standard in detection of carbapenemase production is spectrophotometric measurement of carbapenem hydrolysis in the presence or absence of inhibitor, but it is still reserved for reference laboratories [19]. Recently, the use of mass spectrometry (MALDI-TOFF) based on analysis of degradation of carbapenem molecule enabled rapid detection of KPC carbapenemase (in 45 minutes) or MBL (in 150 minutes) [20, 21]. Finally, simplex or multiplex PCR, real-time PCR, or hybridization tests could significantly improve detection of carbapenemase genes in clinical laboratory bypassing the sensitivity and specificity problems with phenotypic tests. However, molecular methods require expensive equipment and trained laboratory staff.

There are still debates in optimizing possible treatment approach in infections caused by carbapenemase producing strain. It is strongly suggested that combination therapy, including colistin, tigecycline, aminoglycosides, aztreonam, and carbapenems in different combination schemes, is still superior to monotherapy and that carbapenem-containing regimens were superior to others when appropriate dose is applied [17].

Controlling transmission of resistant microorganisms in healthcare setting, which includes carbapenem resistant Enterobacteriaceae (CRE), has several steps. It is important to recognize these bacteria as epidemiologically significant, to know the prevalence in specific region, to be able to identify infected and colonized patients, and to implement measures for stopping the transmission of CRE [22].

There is a bundle of measures which are usually implemented. These include proper hand hygiene, contact isolation, education, strict use of devices, cohorting of patients and staff, laboratory notification, antimicrobial stewardship, and different screening strategies. The best results are achieved only when all measures are simultaneously implemented [23]. Screening of patients at risk is crucial for control of CRE spreading. Screening can be restricted to contacts or to patients that were previously hospitalized in CRE positive institutions. Samples which are usually taken are rectal swabs, stool, or urine. Environmental samples are not useful except for control of disinfection and cleaning. Microbiological laboratory must have guidelines for CRE detection and procedures for rapid notification of CRE positive results. Guidelines from CDC and HICPAC (Healthcare Infection Control Practices Advisory Committee) suggest searching in laboratory data for unrecognized CRE. If positive CRE are found, it is advised to do the point prevalence study on specific departments. After that, it is suggested to perform active surveillance till negative results are obtained. It is necessary to monitor resistance to carbapenems in acute healthcare settings and in long-term care facilities [24].

In conclusion, facing the global crisis in antibiotic resistance, presented by rapid dissemination of carbapenemase producing Gram-negative bacteria, many issues remain controversial, especially detection methods and treatment options. However, active surveillance, hand hygiene, contact precautions, and appropriate antibiotic usage are part of effective approach in reducing incidence of colonization and infections caused by these life treating microorganisms.

*Branka Bedenić* *Vanda Plečko* *Sanda Sardelić* *Selma Uzunović* *Karmen Godič Torkar*
